# Gender Differences and the Impact of Partnership and Children on Quality of Life During the COVID-19 Pandemic

**DOI:** 10.3389/ijph.2023.1605826

**Published:** 2023-05-22

**Authors:** Nora Hettich-Damm, Juila Petersen, Daniela Zahn, Rieke Baumkoetter, Philipp S. Wild, Thomas Muenzel, Andreas K. Schuster, Jochem Koenig, Karl Lackner, Norbert Pfeiffer, Manfred E. Beutel, Elisabeth Engwicht

**Affiliations:** ^1^ Department of Psychosomatic Medicine and Psychotherapy, University Medical Center of the Johannes Gutenberg University Mainz, Mainz, Germany; ^2^ Center for Cardiology, University Medical Center of the Johannes Gutenberg University Mainz, Mainz, Germany; ^3^ Partner Site Rhine-Main, DZHK, German Center for Cardiovascular Research, Bad Nauheim, Germany; ^4^ Institute of Molecular Biology, University Medical Center of the Johannes Gutenberg University Mainz, Mainz, Germany; ^5^ Center for Thrombosis and Hemostasis, University Medical Center of the Johannes Gutenberg University Mainz, Mainz, Germany; ^6^ Department of Cardiology I, University Medical Center of the Johannes Gutenberg University Mainz, Mainz, Germany; ^7^ Eye Clinic and Polyclinic, University Medical Center of the Johannes Gutenberg University Mainz, Mainz, Germany; ^8^ Institute of Medical Biostatistics, Epidemiology and Informatics, University Medical Center of the Johannes Gutenberg University Mainz, Mainz, Germany; ^9^ Institute of Clinical Chemistry and Laboratory Medicine, University Medical Center of the Johannes Gutenberg University Mainz, Mainz, Germany

**Keywords:** gender differences, coronavirus, quality of life, COVID-19 pandemic, partnership

## Abstract

**Objectives:** The COVID-19 pandemic and its protective measures have changed the daily lives of families and may have affected quality of life (QoL). The aim of this study was to analyze gender differences in QoL and to examine individuals living in different partnership and family constellations.

**Methods:** Data from the Gutenberg COVID-19 cohort study (N = 10,250) with two measurement time points during the pandemic (2020 and 2021) were used. QoL was assessed using the EUROHIS-QOL questionnaire. Descriptive analyses and autoregressive regressions were performed.

**Results:** Women reported lower QoL than men, and QoL was significantly lower at the second measurement time point in both men and women. Older age, male gender, no migration background, and higher socioeconomic status, as well as partnership and children (especially in men), were protective factors for QoL. Women living with children under 14 and single mothers reported significantly lower QoL.

**Conclusion:** Partnership and family were protective factors for QoL. However, women with young children and single mothers are vulnerable groups for lower QoL. Support is especially needed for women with young children.

## Introduction

The ongoing COVID-19 pandemic impacted everyday life on a large scale as the measures taken by governments to combat the spread of the virus changed multiple aspects of family life, social interaction, work, and many more aspects of living. While men were more affected by COVID-19 mortality, women, especially those between the ages of 20 and 59, were more likely to be infected and thus at higher risk of adverse long-term health outcomes [1]. The impact of changes in daily life on the mental health and wellbeing of the general population were mostly studied with a focus on distress (depressiveness and anxiety) [2–4]. In Chile it was found that women reported higher rates of the deterioration of mental health and psychological wellbeing than men. Influencing factors were unemployment, loss of income, and an increase in housework and childcare [5]. Exemplary for the mental health of parents, children, and family functioning in the United States, one study found a larger decline in depression in view of the pandemic for mothers relatively to fathers. Female gender and low family income were characterized as risk factors for decline of wellbeing [6]. However, quality of life (QoL)—an important endpoint in public health research—can also function as another key (mental) health indicator for the general population in the midst of the pandemic [7]. Previous studies taking variables as relationship status, children, and single parenthood and their association with QoL and psychological wellbeing during the pandemic into account mostly did not analyze gender differences [8].

Health-related QoL using the EQ-5D was examined in a Portuguese sample focusing on the conditions under the COVID-19 quarantine. It was found that participants who lived with a partner or were married reported higher QoL whereas singles reported the lowest QoL [9]. Changes of QoL during the COVID-19 pandemic were studied in an Austrian sample. Focusing on gender differences, men in a partnership or marriage reported more likely improved QoL compared to single men. In contrast to this, single women reported more likely improved QoL, and women having a partnership or being married were less likely to report an increased quality of life, compared to single women. All participants whether female or male who took care of children (6–16 years) reported more likely decreased QoL compared to individuals without children [10].

A representative online-survey addressed the mental health of families in the Netherlands using a cross-sectional study design to illuminate the effects of lockdown measures and gender on paid work, the division of childcare, and household tasks and quality of life in April 2020. In this study, mothers reported a larger decline of leisure time than fathers. Moreover, mothers were more likely to shift their working hours to evenings or the weekend. Fathers were more likely to report an increase in the relative share of childcare tasks compared to before the pandemic. Parents of younger children (primary school) were more likely to report a decline of leisure-time facing the pandemic than parents of older children. No gender inequalities were found with respect to decline of perceived work-life-balance. Regarding relationship dynamics, disagreements concerning childcare were more likely for parents with small children [11]. First evidence for lower wellbeing of single parents during the COVID-19 pandemic was provided by an Australian online survey revealing associations between lower subjective wellbeing and being a single parent, low education, foreign language, and government benefits [8].

Adding to existing studies based on mainly cross-sectional online surveys with convenience samples, the current study analyzed data from a face-to-face cohort study with additional questionnaires using a representative sample of more than 10,000 individuals with two measurement time points during the pandemic. The aim of this study was to analyze potential differences in quality of life between women and men regarding partnership constellations and family life. Gaining more insight into interactions between gender and quality of life facing the pandemic is particularly important as the vulnerabilities, rights, and duties of women and men of different age groups have become an important societal issue. The study used the EUROHIS-QoL questionnaire which includes health and non-health related aspects of QoL. This study evaluates the following hypotheses:

1) Partnership and having children are protective factors for QoL during the pandemic.
2) Living with younger children and being a single parent are risk factors for low QoL during the pandemic.
3) Women and socially disadvantaged individuals are at higher risk of low QoL.
4) Over the course of the pandemic, women and socially disadvantaged individuals are permanently at risk of lower QoL.

## Methods

### Procedure and Study Sample

The Gutenberg COVID-19 Study (GCS) examines 10,250 individuals including 8,121 from the cohort of the ongoing Gutenberg Health Study (GHS) and 2,129 newly enrolled younger individuals during the pandemic. The GHS is a population-based, prospective, observational, single-center cohort study in the Rhine-Main-Region in Germany. The random sample is stratified by decades of age (25–89 years), gender (male, female), and place of residence (city of Mainz, county of Mainz-Bingen). Sufficient fluency in German and the ability to come to the study center were required. Data from the GCS baseline survey T1 (October 2020 to April 2021) and from the 4-month follow-up T2 (March to June 2021) were analyzed. In the follow-up survey, 9,145 individuals participated.

### Measures

Quality of life was assessed with the EUROHIS-QOL 8 Item Index (EUROHIS-QOL) using a self-report questionnaire [12]. This self-assessment instrument measures general quality of life regarding psychological, physical, social, and environmental life domains. Each domain is represented by two items. The index value is formed by the summation of the 8 item values and higher values indicate better quality of life. The items are rated on a five-point Likert scale (“not at all” to “completely”). Former studies indicated good psychometric properties of the German version of the instrument [13–15].

Group variables and predictors of the current study included [1] different partnership constellations (single vs. living apart vs. living together with the partner) [2], having children vs. having no children [3], being a single parent vs. not being a single parent, and [4] minors living in the household (no minors in the household vs. minors older than 14 years vs. minors younger than 14). Persons living with individuals of different ages were included in the group of the youngest person they live with. Partnership constellations and children were assessed during a standardized personal computer-assisted interviews. The question about partnership constellations was asked in a gender-neutral way. Single parenthood and minors in the household were assessed with self-report questionnaires.

We also included sociodemographic variables such as gender, age, education, and reported migration background, which were all assessed during a standardized personal computer-assisted interview. Additionally, we considered the equivalized household income and socio-economic status using the index of Lampert and Kroll (SES) [16].

### Statistical Analysis

Descriptive analyses were performed as absolute and relative proportions as well as means and standard deviations of the full sample and stratified by gender. Group comparisons between the two genders, the group variables (partnership, children, living with minors, single parents), and measurement times were performed by using t-test and ANOVAs.

To prepare the data for autoregressive modelling, we conducted an exploratory and confirmatory factor analysis and tested for measurement invariance. To identify risk and protective factors for quality of life, we estimated four autoregressive models [17, 18] for the two time points during the pandemic. Autoregressive models assume that each latent construct η _i_ (in this case quality of life) measured at a time t is a function of its prior value at t-1 ( η _it-1_) plus random error ε _it_ [19]. Autoregressive models were conducted because they provide important information about relevant risk and protective factors separately for each time point. By regressing the prior value on the current value of the latent construct, the effects of the explanatory factors on QoL at T2 are estimated, which, in addition to their effects on QoL at T1, have an additional impact on QoL between time points. Sociodemographic predictors for these models were gender, age, migration background, and socioeconomic status. Group predictors were partnership (model 1), having children (model 2), living with minors (model 3), and being a single parent (model 4). All models were estimated using the SEM technique to control for measurement error. To evaluate the best fitted model, the combination of the four model fit indices CFI, TLI, RMSEA, and SRMR was used. All analyses and tests were performed using R (Version 1.3.1093, packages: car, lsr, carData, dplyr, psych, lavaan).

## Results

### Sample Characteristics

Table 1 shows the characteristics of the total sample at follow-up. The mean age was 56.98 years (range 25–89) and the mean equivalized household income was 2,950 € per month. Of the participants, 50.3% were female, 50% had tertiary education, and 21.2% had a migration background.

**TABLE 1:** Sample characteristics, N = 9,145 (Mainz, Germany. 2023).

	Total sample N = 9,145	Men N = 4,546 (49.7%)	Women N = 4,599 (50.3%)	
	M (SD)	M (SD)	M (SD)	*p*-value
Age	56.98 (15.33)	58.42 (15.29)	55.55 (15.23)	**<0.001**
Equivalized household Income	2950.00 (1848.33)	3045.30 (1920.79)	2855.79 (1768.97)	**<0.001**
SES	14.68 (4.20)	15.24 (4.17)	14.15 (4.15)	**<0.001**
	N (%)	N (%)	N (%)	
Migration (yes) N = 9,134	1,946 (21.3%)	938 (20.7%)	1,008 (21.9%)	0.15
Higher Education (yes) N = 7,360	3,681 (50.0%)	2,062 (54.6%)	1,619 (45.2%)	**<0.001**
Partnership N = 7,227				
Single (yes)	1,326 (18.3%)	489 (14.0%)	837 (22.4%)	**<0.001**
Living apart from partner (yes)	577 (8.0%)	270 (7.8%)	307 (8.2%)	0.51
Living with partner (yes)	5,324 (73.7%)	2,724 (78.2%)	2,600 (69.4%)	**<0.001**
Children (yes) N = 7,042	5,700 (77.2%)	2,924 (77.2%)	2,776 (77.2%)	1.00
Minors living in household (hh)				
Minors 0–14 in hh (yes)	1,750 (20.5%)	843 (20.0%)	907 (20.9%)	0.33
Minors 15–17 in hh (yes)	327 (3.8%)	163 (3.9%)	164 (3.8%)	0.87
No minors living in hh (yes)	6,463 (75.7%)	3,199 (76.1%)	3,264 (75.3%)	0.41
Single parent (yes) N = 3,191	143 (4.5%)	33 (2.0%)	110 (7.0%)	**<0.001**
QoL sum score T1	32.20 (4.23)	32.50 (4.09)	31.90 (4.35)	**<0.001**
QoL sum score T2	31.75 (4.42)	32.19 (4.27)	31.33 (4.53)	**<0.001**

Note: SES: socioeconomic status [16]; significant *p*-values in bold.

### Descriptive Analyses

**FIGURE 1:**
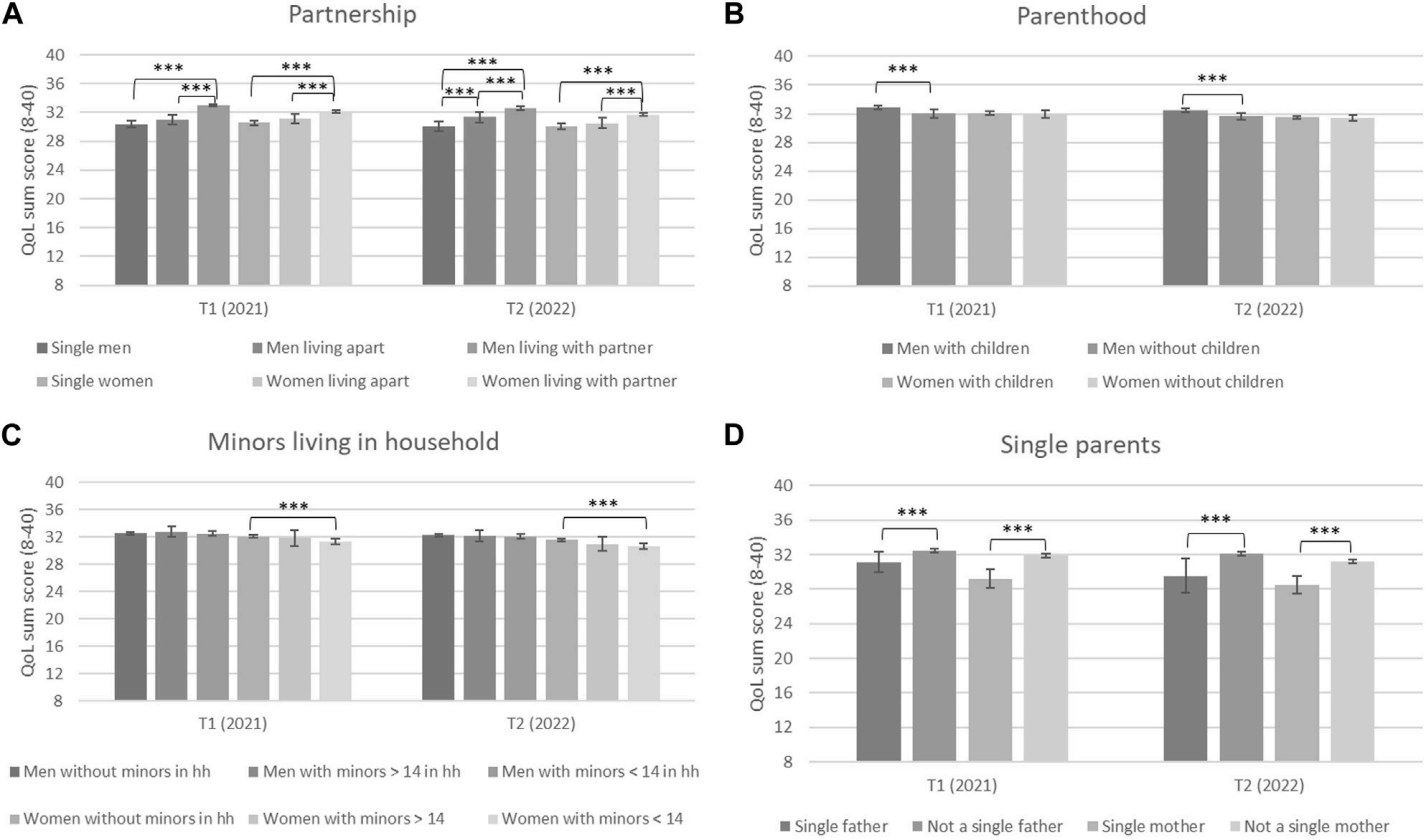
Differences in QoL for men and women living in different partnership **(A)** and family constellations **(B–D)**, presented for T1 and T2, error indicators: CI 95%; *** = p-value < 0.001 (Mainz, Germany. 2023).

Figures 1A–D shows differences in QoL for men and women living in different partnership and family constellations for both time points.

At the first time point in 2020, single men and men who had a partner but lived apart reported a significantly lower QoL sum score than men who lived together with a partner (Figure 1A). There were no significant differences between single men and men who had a partner but did not live together. The same pattern applied for women. At the second time point in 2021, an additional effect was found for men: single men also reported significantly lower QoL scores than men who have a partner but did not live together. Looking at changes over time, men living with a partner and women in each constellation—single, living apart or living together with a partner—reported significantly lower quality of life at T2.

Figure 1B shows differences in QoL for men and women with and without children for both time points. At both time points, men with children reported a significantly higher total QoL score, whereas no significant difference in QoL was found for women with and without children. Looking at changes over time, men and women reported significantly lower QoL at T2, regardless of whether they had children or not.

Figure 1C shows differences in QoL for men and women living with or without minors in their household for both time points. At both time points, no differences in QoL were found for men related to living with or without minors. At both time points, women living without minors in the household reported significantly higher QoL than women living with minors under the age of 14. Looking at changes over time, men and women of all groups reported significantly lower QoL at T2.

Figure 1D shows the differences in QoL between men and women who are single parents for both time points. At both time points, single fathers and mothers reported significantly lower QoL than fathers and mothers who were not single. Looking at changes over time, single fathers and mothers reported no significant changes, while mothers and fathers who were not single reported significantly lower QoL at T2.

### Exploratory Factor Analysis

Table 2 shows the results of the exploratory factor analysis. The Kaiser-Meyer-Olkin (KMO) measure verified the sampling adequacy of the items with the KMO ranging from KMO = 0.85 to KMO = 0.91. Bartlett’s test of sphericity indicated an adequate correlation structure for factor analysis [X^2^ (28) = 248,219, *p* > 0.001]. The principal axis factor analysis with the Kaiser criterion of eigenvalues greater than 1 suggested a two-factor solution. However, “relationship” did not demonstrate a loading on either factor of more than 0.4. This structure was confirmed by a CFA. Therefore, we opted for a hierarchical structural equation model with QoL as a latent higher order variable consisting of an “intrinsic quality of life factor” and an “extrinsic quality of life factor” as lower order factors and excluded the item “relationship” for the analysis of the autoregressive models. Both factors correlated strongly (Phi = 0.68). The intrinsic quality of life factors dimension exhibited a Cronbach’s alpha of a = 0.86; the extrinsic quality of life factors dimension exhibited a Cronbach’s alpha of a = 0.62 (for T1). The full QoL-scale (excluding “relationship”) yields a Cronbach’s alpha of a = 0.82.

**TABLE 2:** Exploratory factor analysis (Mainz, Germany. 2023).

Item	Factor 1	Factor 2	Dimension
Health state	0.62	0.01	Intrinsic factors of QoL
Energy	0.81	−0.03	
Daily routine	0.84	−0.09	
Self	0.78	0.00	
Relationships	**0.32**	**0.28**	
Livability	0.32	0.40	Extrinsic factors of QoL
Money	−0.05	0.59	
Housing	−0.08	0.69	
Eigenvalue	2.60	1.10	
Variance	0.33	0.14	
Cumulative variance		0.49	

Note: Extraction method: principal axis, rotation method: promax oblique rotation.

loadings of the excluded item in bold.

The test for measurement invariance, overall, yielded very good fits for the bifactor model (Table 3) with CFI > 0.96, TLI > 0.95, SRMR < 0.08, and RMSEA < 0.06 [20, 21]. Since a change in CFI ≤ 0.01 is considered acceptable [22] it can be concluded that strict measurement invariance is given and the means between the time points can be interpreted as reflective of real differences and a result of underlying factors (e.g., gender).

**TABLE 3:** Fit indices for testing for measurement invariance (Mainz, Germany. 2023).

	CFI	△ CFI	TLI	△ TLI	RMSEA	△ RMSEA	SRMR	△ SRMR
Configural	0.967		0.954		0.060		0.044	
Weak	0.967	0.000	0.958	0.004	0.058	−0.002	0.046	0.002
Strong	0.965	−0.002	0.957	−0.001	0.058	0.000	0.047	0.001
Strict	0.963	−0.002	0.959	0.002	0.057	−0.001	0.048	0.001

Note: CFI, Comparative Fit Index; TLI, Tucker-Lewis Index; RMSEA, Root Meant Square Error of Approximation; SRMR, standardized root mean square residual.

### Autoregressive Models

Table 4 shows autoregressive models for the sum score of QoL (item *relationship* excluded) with sociodemographic predictors (model 1) as well as partnership (model 2), parenthood and minors living in the household (model 3), and single parents (model 4). In the longitudinal perspective, the total effects of the autoregressive models showed that male gender, higher age, no migration background, and a higher SES predicted higher QoL (model 1). In model 2, gender was not predictive anymore, but both living together and living apart from a partner were associated with higher QoL. Additionally, having children was predictive for higher QoL, but being a woman and living with children younger than 14 years old was associated with lower QoL (model 3) as well as being a single mother (model 4). The strongest predictor in model 3 was SES followed by living with a partner, being a woman who lives with minors under 14 years, age, living apart from the partner, children, and migration background.

**TABLE 4:** Autoregressive models for the sum score of QoL with sociodemographic predictors as well as partnership, parenthood, and minors in the household (Mainz, Germany. 2023).

	Model 1—sociodemographic				Model 2—partnership				Model 3—parenthood and minors in household				Model 4—Single parents			
	N = 8,377				N = 6,879				N = 5,311				N = 1,726			
Fit Measures	Standard		Robust		Standard		Robust		Standard		Robust		Standard		Robust	
	CFI = 0.922		CFI = 0.927		CFI = 0.920		CFI = 0.923		CFI = 0.929		CFI = 0.931		CFI = 0.926		CFI = 0.928	
	TLI = 0.899		TLI = 0.905		TLI = 0.899		TLI = 0.903		TLI = 0.912		TLI = 0.915		TLI = 0.910		TLI = 0.912	
	RMSEA = 0.064		RMSEA = 0.069		RMSEA = 0.056		RMSEA = 0.060		RMSEA = 0.045		RMSEA = 0.047		RMSEA = 0.044		RMSEA = 0.046	
	SRMR = 0.054		SRMR = 0.054		SRMR = 0.050		SRMR = 0.050		SRMR = 0.040		SRMR = 0.040		SRMR = 0.042		SRMR = 0.042	
	beta	se	z	p	beta	se	z	p	beta	se	z	p	beta	se	z	p
Total effects																
Gender (women)	−0.095	0.013	−7.348	**<0.001**	−0.041	0.035	−1.196	0.232	−0.007	0.047	−0.156	0.876	0.085	0.119	0.734	0.463
Age	0.115	0.000	9.095	**<0.001**	0.110	0.000	7.992	**<0.001**	0.071	0.001	3.835	**<0.001**	−0.003	0.002	−0.090	0.928
Migration background (yes)	−0.043	0.015	−3.601	**<0.001**	−0.047	0.017	−3.552	**<0.001**	−0.043	0.019	−2.943	**0.003**	−0.045	0.033	−1.771	0.077
SES	0.165	0.002	11.532	**<0.001**	0.137	0.002	8.562	**<0.001**	0.155	0.002	8.708	**<0.001**	0.150	0.004	4.789	**<0.001**
Partner living apart (ref.: single)					0.055	0.047	2.253	**0.024**	0.063	0.054	2.401	**0.016**	−0.016	0.116	−0.307	0.759
Living apart x gender					−0.019	0.064	−0.764	0.445	−0.047	0.076	−1.709	0.088	0.038	0.151	0.742	0.458
Partner living together (ref.: single)					0.165	0.030	6.526	**<0.001**	0.156	0.036	5.288	**<0.001**	0.048	0.094	0.787	0.431
Living together x gender					−0.064	0.038	−1.801	0.072	−0.036	0.045	−0.890	0.373	−0.093	0.120	−0.821	0.411
Children (yes)									0.052	0.028	2.303	**0.021**	0.052	0.054	1.349	0.177
Children x gender									−0.017	0.039	−0.493	0.622	−0.074	0.049	−0.996	0.319
Minors <14 in hh (ref.: no minors)									−0.040	0.033	−1.765	0.078	−0.064	0.047	−1.418	0.156
Minors <14 x gender									−0.076	0.046	−3.326	**0.001**	−0.116	0.063	−2.354	**0.019**
Minors >14 in hh (ref.: no minors)									−0.024	0.050	−1.238	0.216	−0.036	0.059	−1.039	0.299
Minors >14 x gender									−0.024	0.075	−1.134	0.257	0.001	0.090	0.034	0.973
Single Parents (yes)													−0.002	0.115	−0.036	0.971
Single Parents x gender													−0.111	0.148	−2.011	**0.044**
QoL T1∼																
Gender (women)	−0.060	0.013	−4.603	**<0.001**	−0.037	0.034	−1.058	0.290	0.021	0.046	0.433	0.665	0.062	0.115	0.492	0.623
Age	0.169	0.000	13.286	**<0.001**	0.174	0.000	12.580	**<0.001**	0.109	0.001	5.694	**<0.001**	0.046	0.001	1.320	0.187
Migration background (yes)	−0.036	0.015	−2.987	**0.003**	−0.039	0.016	−2.928	**0.003**	−0.037	0.018	−2.482	**0.013**	−0.054	0.030	−2.058	**0.040**
SES	0.218	0.002	14.994	**<0.001**	0.178	0.002	10.981	**<0.001**	0.196	0.002	10.748	**<0.001**	0.202	0.004	6.281	**<0.001**
Partner living apart (ref.: single)					0.008	0.046	0.318	0.750	0.043	0.052	1.521	0.128	−0.087	0.115	−1.496	0.135
Living apart x gender					0.033	0.062	1.260	0.208	−0.023	0.072	−0.806	0.420	0.099	0.152	1.676	0.094
Partner living together (ref.: single)					0.157	0.028	6.209	**<0.001**	0.166	0.033	5.599	**<0.001**	0.059	0.087	0.905	0.366
Living together x gender					−0.038	0.036	−1.037	0.300	−0.026	0.043	−0.610	0.542	−0.038	0.112	−0.319	0.750
Children (yes)									0.032	0.026	1.371	0.170	0.046	0.048	1.179	0.238
Children x gender									−0.030	0.036	−0.816	0.415	−0.062	0.073	−0.785	0.432
Minors <14 in hh (ref.: no minors)									−0.025	0.030	−1.123	0.261	−0.044	0.042	−0.970	0.332
Minors <14 x gender									−0.071	0.042	−3.131	**0.002**	−0.124	0.056	−2.484	**0.013**
Minors >14 in hh (ref.: no minors)									−0.030	0.044	−1.591	0.112	−0.026	0.051	−0.769	0.442
Minors >14 x gender									−0.003	0.070	−0.157	0.875	0.014	0.082	0.339	0.735
Single Parents (yes)													−0.038	0.100	−0.777	0.437
Single Parents x gender													−0.075	0.137	−1.288	0.198
QoL T2∼																
QoL T1	0.893	0.023	41.344	**<0.001**	0.893	0.026	37.340	**<0.001**	0.888	0.030	32.859	**<0.001**	0.894	0.055	18.623	**<0.001**
Gender (women)	−0.041	0.009	−4.801	**<0.001**	−0.008	0.024	−0.326	0.744	−0.025	0.031	−0.856	0.392	0.029	0.082	0.364	0.716
Age	−0.036	0.000	−3.942	**<0.001**	−0.045	0.000	−4.408	**<0.001**	−0.026	0.001	−2.076	**0.038**	−0.044	0.001	−1.794	0.073
Migration background (yes)	−0.011	0.011	−1.267	0.205	−0.012	0.012	−1.295	0.195	−0.010	0.013	−0.961	0.336	0.003	0.022	0.184	0.854
SES	−0.029	0.001	−2.892	**0.004**	−0.022	0.001	−2.040	**0.041**	−0.019	0.002	−1.510	0.131	−0.031	0.003	−1.368	0.171
Partner living apart (ref.: single)					0.048	0.032	2.880	**0.004**	0.026	0.036	1.461	0.144	0.062	0.092	1.522	0.128
Living apart x gender					−0.049	0.043	−2.898	**0.004**	−0.027	0.050	−1.458	0.145	−0.051	0.108	−1.362	0.173
Partner living together (ref.: single)					0.025	0.020	1.465	0.143	0.009	0.024	0.477	0.633	−0.004	0.064	−0.100	0.920
Living together x gender					−0.031	0.026	−1.243	0.214	−0.014	0.030	−0.497	0.619	−0.059	0.084	−0.744	0.457
Children (yes)									0.024	0.019	1.563	0.118	0.011	0.037	0.413	0.679
Children x gender									0.009	0.026	0.381	0.703	−0.019	0.051	−0.385	0.700
Minors <14 in hh (ref.: no minors)									−0.018	0.022	−1.174	0.241	−0.025	0.031	−0.844	0.399
Minors <14 x gender									−0.012	0.032	−0.788	0.431	−0.005	0.042	−0.148	0.882
Minors >14 in hh (ref.: no minors)									0.002	0.037	0.153	0.878	−0.013	0.044	−0.493	0.622
Minors >14 x gender									−0.021	0.056	−1.319	0.187	−0.011	0.068	−0.374	0.708
Single Parents (yes)													0.032	0.075	1.001	0.317
Single Parents x gender													−0.044	0.097	−1.220	0.223

Note: SES, socioeconomic status [16]; hh, household; significant *p*-values in bold.

Cross sectional analysis showed a quite similar picture for T1. Older people, individuals without migration background, and persons with a higher SES reported higher QoL (model 1). Additionally, individuals living together with a partner reported higher QoL (model 2) and women living with minors younger than 14 years old reported lower QoL (model 3). In model 3 the strongest predictor was SES followed by living with a partner, age, women living with minors younger than 14 years, and migration background.

For T2, the strongest predictor was QoL at T1. Additionally, being male, younger, and having a lower SES were significant predictors for higher QoL in model 1. Age and SES stayed predictive in model 2, were also living apart from a partner was associated with higher QoL. However, women living apart from their partner reported significantly lower QoL at T2. In model 3, only younger age and QoL at T1 were significant predictors.

## Discussion

This study analyzed QoL of women and men at two time points during the COVID-19 pandemic focusing on partnership and family life. In 2020 and 2021, men and women differed significantly in their QoL with women reporting a lower sum score. This is in line with publications concerning QoL in men and women in the German population [13–15]. Our results also show, as one of the first longitudinal analysis during the pandemic, that quality of life as measured by the EUROHIS-QOL was significantly lower at the second measurement time point during the pandemic for both men and women, indicating that the ongoing pandemic and its measures were increasingly stressful for the general German population.

Regarding socio-economic predictors in the longitudinal perspective, older individuals, persons without migration background, and with a higher SES reported higher QoL. This is partly in line with previous studies which identified migration background as a risk factor for QoL [15, 23–25]. Moreover, higher SES was previously associated with higher QoL [15, 26]. Thus, as hypothesized especially socially disadvantaged individuals showed lower QoL. Contrary to other findings [13, 15] before the pandemic, older people reported higher QoL. This might be a selection effect as the individuals in our sample were relatively old on average (M = 56.98) and had a relatively high SES (M = 14.68) in comparison to the general German population [15, 27, 28]. Another explanation could be that younger people are more troubled by the pandemic and its safety measurements and therefore reported lower QoL during the pandemic as some other studies found [15, 29, 30].

Concerning partnership, in line with the hypothesis and previous studies [9], the results showed that during the pandemic living together with a partner was a protective factor for QoL for men and women. However, this is in contrast to studies indicating that only men who were in a partnership or married reported improved QoL during the pandemic [10]. In the longitudinal perspective, both living together and living apart from a partner were associated with higher QoL. Presumably, having a partner could be associated with less loneliness and therefore higher QoL. Although partnership is a protective factor before and during the pandemic, in our study QoL decreased during the pandemic for all women regardless of their partnership constellation and for men living together with a partner. Nevertheless, the differences reported at T1 were quite large between men living together with a partner and men living apart or being single. These differences in QoL were still significant at T2 although QoL decreased significantly for men living with a partner.

Regarding families, the results indicated gender inequalities. In contrast to other studies [10], having children was positively associated with QoL in the longitudinal perspective. Probably, many individuals from the sample had older children who served as social support during the difficult times of lockdown and uncertainty. In general and during the pandemic, social support was found to be a protective factor for high QoL [31, 32]. However, descriptive analysis showed that only men having children reported significantly higher QoL compared to men without children. This might be seen as an indicator for unequal responsibilities in childcare during the pandemic. Presumably, men profited from more time at home promoting family cohesion and having more time for interaction with the family, while women were responsible to organize the family during the difficult phases of lockdowns [11]. This suggestion was underlined by the result that only women living with minors under 14 years of age reported lower QoL. Additional parental supervision and even help with homeschooling needed during the lockdown might above all burden women [33]. The positive effect of having children on quality of life during the pandemic was especially visible for men who presumably profit from more social support and appreciation in face of childcare tasks than women.

Those findings indicate, as hypothesized, that women with small children are more burdened by the pandemic and its protective measurements. As previous studies found, childcare work is still more done by women than by men which was reinforced by the pandemic [33–36]. In the longitudinal perspective, the same was true for single parents as the regression showed a significant interaction effect between single parent and gender with single mothers reporting lower QoL. However, in the descriptive analysis, single fathers and single mothers reported lower QoL compared to families with two parents. Although the subsample of single parents was quite small, other studies hinted in the same direction [8, 37] indicating that this group of parents needs to be explored further.

### Strengths and Limitations

Benefits of the study were the availability of data of a representative sample. It also highlighted that QoL is a strong indicator for daily life of families that has previously not been widely studied during the pandemic. However, the results need to be interpreted considering the study’s limitations. Two measurement times during the pandemic were analyzed while we had no data before the pandemic. Moreover, the time to follow-up was only 4 months. Since the mean age of our sample is relatively high (M = 56.98; SD = 15.33), the average quality of life could also be higher than in younger samples, considering that older individuals generally report a higher quality of life [15]. The sample size of single parents, especially single fathers was small. Employment was not considered as an influencing factor in the analysis. However, the pandemic showed relevant effects on employment, which also differed between women and men [38]. This could be related to quality of life and should be considered in a further project on quality of life during the pandemic. The sexual orientation of the participants was not assessed and it is not possible to conclude anything about partnerships or parenthood of individuals with different sexual orientations. However, it was not assumed that all participants were heterosexual, since the question about partnership constellations was asked in a gender-neutral way.

### Conclusion

During the pandemic, QoL was lower in women than in men and decreased over the course of the pandemic. Partnership as well as having children were found to be a protective factor of QoL during the pandemic. However, especially women with small children and single mothers reported lower QoL and seemed to be burdened the most. As QoL is a valuable indicator for mental health also during the pandemic [32], it seems important to support women with children during the pandemic. Moreover, the involvement of fathers into childcare and family tasks might be important to promote gender equality.
